# Confidence as a Common Currency between Vision and Audition

**DOI:** 10.1371/journal.pone.0147901

**Published:** 2016-01-25

**Authors:** Vincent de Gardelle, François Le Corre, Pascal Mamassian

**Affiliations:** 1 Centre d’Economie de la Sorbonne, CNRS UMR 8174, Paris, France; 2 Paris School of Economics, Paris, France; 3 Laboratoire des Systèmes Perceptifs, CNRS UMR 8248, Paris, France; 4 Institut d’Etude de la Cognition, École Normale Supérieure—PSL Research University, Paris, France; Centre de Neuroscience Cognitive, FRANCE

## Abstract

The idea of a common currency underlying our choice behaviour has played an important role in sciences of behaviour, from neurobiology to psychology and economics. However, while it has been mainly investigated in terms of values, with a common scale on which goods would be evaluated and compared, the question of a common scale for subjective probabilities and confidence in particular has received only little empirical investigation so far. The present study extends previous work addressing this question, by showing that confidence can be compared across visual and auditory decisions, with the same precision as for the comparison of two trials within the same task. We discuss the possibility that confidence could serve as a common currency when describing our choices to ourselves and to others.

## Introduction

When an individual agent faces a choice between several options, having a common currency to evaluate all options seems useful for comparing them and selecting the best one. One illustration is the euro effect in the domain of international economics: introducing euro as a common currency greatly increased trading within the euro zone (for a meta-analysis see [1]). Switching to euro facilitated potential transactions for agents, by reducing the costs and uncertainties related to conversions between different currencies.

At the level of individual agents, decision theory has always relied on the notion of utility [2,3] that is, a common scale on which options can be compared, in order to select the option that maximizes utility. Similarly, the idea that the brain needs a common currency to select between several courses of action (i.e. getting the apple or the orange, sleeping or hunting) has been put forward in the field of behavioural biology, with e.g. reproductive value [4] or pleasure [5] being proposed as common currencies. More recently, neuroimaging studies showing the involvement of the ventro-medial prefrontal cortex in the valuation of diverse types of goods have provided empirical support to this notion of a common utility scale [6,7].

However, when computing subjective expected utility [2], the agent needs not only to have a common utility scale but also a common probability scale between the different options. In other words, the agent should be able to compare subjective probabilities (i.e. beliefs) associated with diverse types of events. This idea that subjective probabilities involve a common scale has received much less direct scrutiny than the idea of a common value scale (although see [8]).

In the present study, we focus more specifically on one particular type of subjective probability that is post-decision confidence. Post-decision confidence corresponds to the agent’s subjective probability that the just-made decision was correct. Earlier findings that overconfidence (i.e. the overestimation of one’s own probability of success) are correlated across a knowledge test and a motor task [9], or across a memory task and a knowledge task [10] seem consistent with the idea that confidence is represented on a common scale for various types of events, with the same precision and the same biases. Recent studies investigating the relation between confidence in memory and perceptual decisions provided mixed results [11,12]. Thus, the full extent to which this generality of confidence holds across any domains is still a matter of empirical research.

In a previous study [13], we addressed more directly the possibility that confidence might involve a common scale across different decision tasks. To do so, we reasoned that if confidence was encoded in a task-specific format, then it should be more comparable across two trials involving the same task than across two trials involving different tasks. If, however, confidence was readily available under a task-generic format, i.e. if confidence involved a common scale across the two tasks, then the within-task comparison and the across-tasks comparison should be equivalent. In this previous study, we used orientation and spatial frequency discrimination tasks on visual stimuli (Gabor patches), and we found that participants’ confidence comparisons were equally good in within-task conditions and across-tasks conditions. This finding provided empirical support for the idea that confidence involves a common scale between these two visual tasks.

In the present study, our objective is to probe further this idea of a common scale for confidence, by testing whether this common scale holds even across distinct sensory modalities, namely vision and audition. This test is important because there are reports that comparisons across sensory modalities behave differently than within modalities [14]. We employed the same methodology as in our previous study, and replaced the visual spatial frequency task with the auditory task of pitch discrimination between two pure tones.

## Methods

### Participants

Twenty-four volunteers were recruited via the French RISC database (http://www.risc.cnrs.fr). This number was chosen by design for counterbalancing the order of the 4 experimental blocks. They all provided informed consent and received 15 euros. Four participants were discarded from all analyses: 2 showing extremely high performance in the auditory task (sensitivity greater than the group average by more than 2 SD), 1 showing chance performance in the visual-visual block, and 1 showing a strong bias in the confidence comparison task (favoring audition in 86% of the across-task comparisons).

### Ethics statement

Written informed consent was obtained from all participants before the experiment. The research was non-invasive; it involved negligible risks and no collection of nominative/identifying information. No health information was collected from participants. Under French regulations, the legal ethics committees do not examine non-invasive behavioral studies. Thus, ethics approval was not required and no IRB was consulted before conducting the study.

### Perceptual tasks (Fig 1)

For the visual trial, two Gabor patch stimuli were presented in succession (inter-stimulus interval: 500ms), and the observer had to judge whether the second stimulus was rotated “clockwise” or “counterclockwise” relative to the first stimulus. Stimuli were about 5° large (in visual angle), they were presented at 40% contrast on a gray background, for 100ms. Spatial frequency was 1.4 cycles per degree. The orientation of the first stimulus was roved (orientation: 4° around vertical), and the orientation of the second stimulus was determined in each block by two randomly interleaved staircases, one that aimed at 80% of “clockwise” responses and one that aimed at 80% of “counterclockwise” responses. We used separate staircases for the first trials and second trials in each pair. For the auditory task, we used 200ms pure tones separated by 200ms silence. Observers had to judge whether the second tone was going “up” (higher pitch) or “down” (lower pitch) relative to the first tone. The pitch of the first stimulus was roved (3 semi-tones around 440Hz) and the pitch of the second stimulus was determined by staircases, as for the visual task. Observers reported their responses on a keyboard, and during the trial we presented visual symbols on the screen to indicate the mapping (fixed within each block) between the stimulus categories and the response keys on the keyboard. Responses were not speeded and no feedback was given.

**Fig 1:**
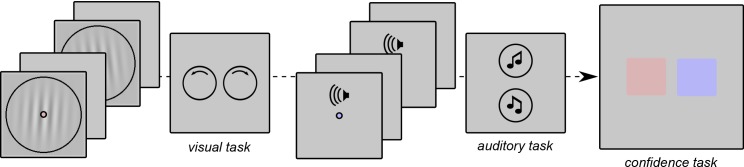
Experimental tasks. We illustrate here a pair of trials for the confidence comparison across tasks. The observer makes an orientation judgment on Gabor patch stimuli (visual task), and then a pitch judgment on pure tones (auditory task). Note that the two tasks are associated with different colors of the fixation point. Finally the observer compares these two decisions in terms of confidence (metacognitive task), and indicates the color of the trial associated with the highest confidence.

### Metacognitive task

For each pair of trials, one was associated with the blue color and the other with the red color (in a random order). This was done by turning the fixation point to red or blue after the stimulus presentation. After each pair of trials, thus, observers had to indicate the color associated with the trial in which they were more confident about their response. The point of this procedure was to minimize the potential response biases in the confidence comparison task.

Design: After being trained separately with the visual task and the auditory task, participants proceeded to the main experiment, which was divided in 4 blocks. Each block consisted in 100 pairs of trials, and the task sequence for all pairs was announced at the beginning and kept constant within the block (either visual-visual or visual-auditory, auditory-visual, auditory-auditory), to minimize task-switching costs [15]. All participants completed all 4 possible blocks, and the ordering of the 4 blocks was counterbalanced across participants.

### Psychophysical analyses

We evaluated the perceptual sensitivity of an observer in a set of trials by means of a psychometric curve. For the visual task, this curve plots the proportion of “counterclockwise” responses as a function of the difference in degrees between the two visual orientations. For the auditory task, the curve plots the proportion of “up” responses against the difference in semitones between the two auditory tones. We fitted these curves by a cumulative Gaussian, of which the inverse standard deviation stands for the sensitivity of the observer for the set of trials under consideration. Fitting was done by minimizing mean-squared error, using Matlab glmfit function with a probit link. In particular, we can assess the sensitivity of perceptual responses for trials associated with higher or lower confidence, as per the confidence comparison responses given by an observer.

### Response time analyses

To avoid contamination by outlying values, we discarded responses faster than 100ms or slower than 3s (less than 10% of the data), and then used the median response time for the remaining data. In a more detailed analysis, we separated the influence of the stimulus on RTs as follows (details of the procedure can be found in the supplementary materials of our previous study [13]). First, we normalized the stimulus values by considering the signed distance S between the stimulus and the point of subjective equality, in standard-deviation units of the psychometric curve, for each participant, confidence level, block type and task. Second, for each of these conditions we grouped the normalized stimulus values in 6 bins, and calculated a median RT for each bin. Third, across 6 bins and 2 confidence levels, we fitted the median RTs with the sum of 3 terms (see equation below): a general intercept A_1_, the effect of S (in units of standard deviation) on RTs with amplitude A_2_, and the residual effect of confidence C (coded as 1 for High Confidence and 0 for Low Confidence) on RTs with amplitude A_3_.

$$RT=A_{1}+A_{2}\times e^{-\frac{1}{2}S^{2}}+A_{3}\times C$$

These 3 parameters (A_1_, A_2_, and A_3_) were estimated separately in each task (visual vs. auditory) x comparison type (within vs. across) condition, for each participant, by minimizing the mean squared error between the predicted and observed data, under the constraints for each parameter (A_1_ in [0, 1], A_2_ in [-1, 1], and A_3_ in [-1, 1]) using Matlab fminsearchbnd function.

All tests were two-sided, unless mentioned otherwise.

## Results

**Fig 2:**
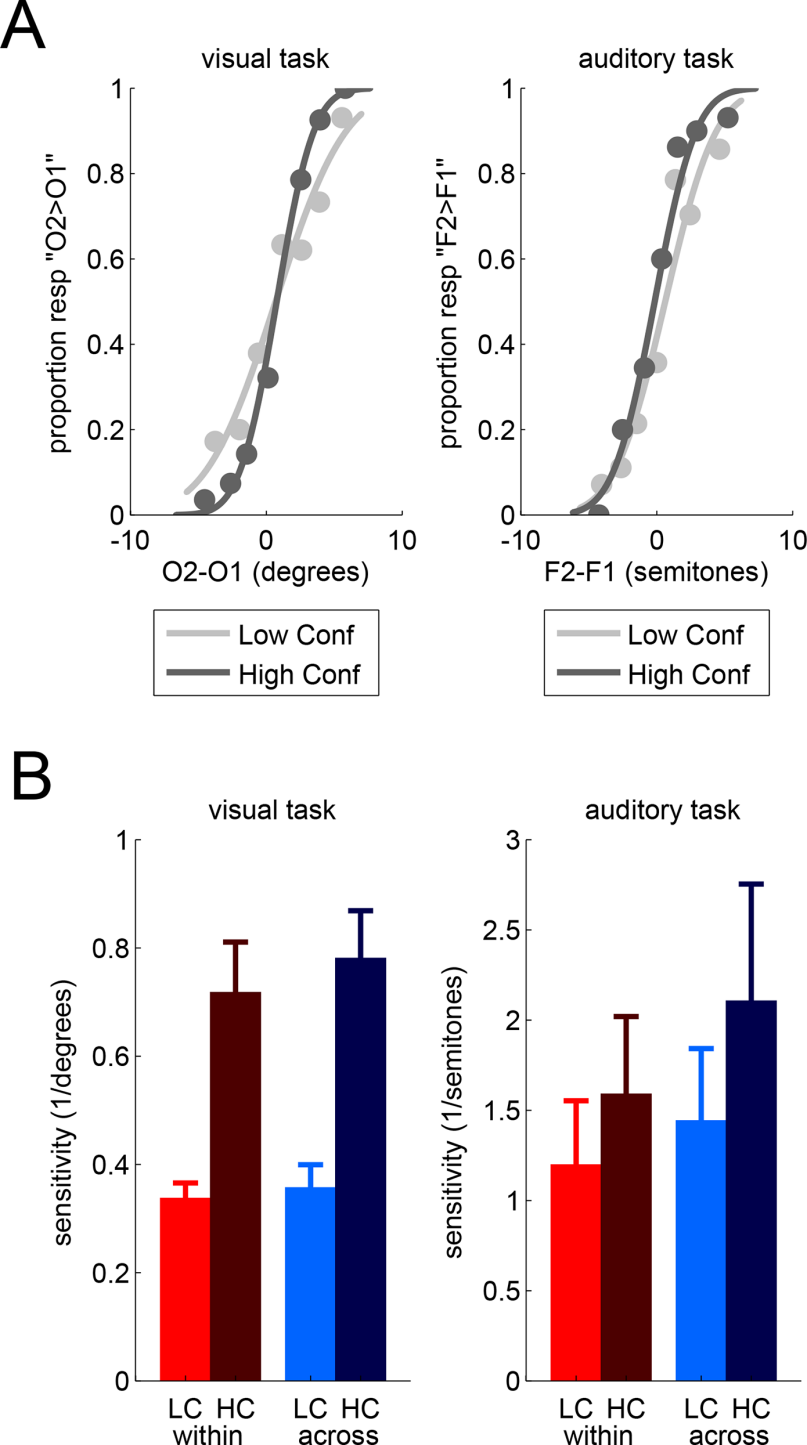
Psychophysical analyses. (A) Psychometric curves for one participant in the visual task (left panel) and in the auditory task (right panel), for trials associated with higher confidence (dark grey) vs. lower confidence (light grey). Perceptual sensitivity is the inverse standard deviation of the cumulative Gaussian fit. (B) Perceptual sensitivity as a function of confidence (LC = low confidence in bright vs. HC = high confidence in dark), confidence comparison conditions (within-task in red vs. across-tasks comparison in blue), and task (visual in the left vs. auditory in the right panel). Bars represent the mean of observers with error bars for the SEM across observers.

For each perceptual task, we evaluated the sensitivity of our observers in subsets of trials defined by 2 factors: confidence level and confidence comparison type. We define the confidence level by means of the comparison response: for each pair of trials, one is associated with higher confidence (*HC condition*) and the other one is associated with lower confidence (*LC condition*). We expect that sensitivity would be higher in trials associated with higher confidence (see Fig 2A). In addition, we define the confidence comparison type as whether the two trials to be compared involve the same task (*within-task condition*) or two different tasks (*across-task condition*).

### Preliminary checks

We verified that our staircase procedures successfully achieved to maintain similar levels of accuracy, irrespective of the task (visual vs. auditory) and the type of block (within vs. across). Indeed, an ANOVA on accuracy with task and block type as within-participant factors indicated no significant main effect or interaction (all p>0.05). The average accuracy across participants was equal to 80% or 81% in all cases. In terms of confidence comparison, our design ensured that we have equal numbers of low confidence and high confidence trials in the within-task condition, for both tasks. However, this balance was not ensured in the across-task condition. As mentioned above, we discarded one participant reporting greater confidence in the auditory task in 86% of the across-task comparisons, leaving only 28 trials in the high-confidence visual condition. On average across the participants included in the analyses, we had 103 trials in the high-confidence auditory and in the low-confidence visual conditions, and 97 trials in the complementary high-confidence visual and low-confidence auditory conditions, with at least 62 trials in each case for each participant. Thus, the imbalance was relatively small on average and not problematic for our subsequent analyses of sensitivity based on psychometric curves for each task, confidence level, and confidence comparison type.

### Metacognitive abilities

On the basis of psychometric curves, we computed measures of sensitivity for each participant. For each task, we ran an analysis of variance (ANOVA) on these measures of sensitivity with confidence level and confidence comparison type as within-participant factors (see Fig 2B). In both the visual and the auditory task, we found a main effect of confidence level (visual: F(1,19) = 31.81, p<0.001; auditory: F(1,19) = 9.68, p = 0.006), by which participants exhibited greater sensitivity in trials associated with higher confidence, as expected. In other words, participants can monitor some fluctuations of their own sensitivity in their confidence comparison reports, thereby showing a form of metacognitive ability. The ANOVAs indicated no main effects of confidence comparison type on sensitivity (both p>0.146) and, importantly, no interaction between confidence level and confidence comparison type (both p>0.529). This absence of interaction is of particular interest, as it suggests that the metacognitive ability was unchanged between the within-task and the across-tasks conditions for confidence comparison.

To quantify more formally the evidence for the absence of an interaction, we turned to analyses of the Bayes Factor (hereafter BF). In a first approach, we found that the probability that there was no difference between the within and across conditions (null hypothesis H0) was relatively high for both tasks (visual: BF = 3.6, p(H0) = 0.78; auditory: BF = 3.7, p(H0) = 0.79). Moreover, the data suggest, if anything, that the difference between low and high confidence conditions seemed more pronounced in the across-task condition, contrary to the alternative hypothesis that confidence is encoded in a task-specific format, which predicts a greater metacognitive ability in the within-task comparison condition. We thus conducted a second Bayes factor computation, to take into account the directionality of the alternative hypothesis [16]. This second analysis provided stronger evidence for the null hypothesis, as expected (visual: BF = 6.8, p(H0) = 0.87; auditory: BF = 6.5, p(H0) = 0.87).

To compare and combine the data across the visual and auditory tasks, we calculated a modulation index, quantifying metacognitive ability as the change in sensitivity from the low confidence to the high confidence condition, relative to the average of the two conditions. For the visual task, the average modulation index was 62% in the within condition and 71% in the across condition; for the auditory task, the average modulation index was 39% in the within condition and 30% in the across condition. T-tests across participants confirmed that modulation indices were positive in all conditions (all p<0.01).

A within-participant ANOVA on these modulation indices indicated that the main effect of task was significant (F(1,19) = 10.89, p = 0.004), with no main effect of type (F(1,19) = 0.0, p = 0.995) and no interaction (F(1,20) = 0.93, p = 0.347). In other words, participants exhibited lower metacognitive abilities in the auditory task (as assessed by our modulation index) than in the visual task, but there was no difference between the within-task and across-task comparisons. The calculation of Bayes Factors (using the correction for the directionality of the alternative hypothesis) provided evidence for the null hypothesis H0 (no difference in the modulation indices between the within-task and the across-tasks comparisons), both in the visual task (BF = 7.6, p(H0) = 0.88) and in the auditory task (BF = 2.5, p(H0) = 0.71) in isolation, but also when the two tasks were considered together by averaging the modulation indices across tasks for each participant (BF = 4.3, p(H0) = 0.81).

### Response times

Finally, we assessed potential differences between the within-task and across-task blocks in terms of response times (RTs). Median RTs for the confidence comparison judgments were slightly longer for in the across-task comparison (M = 650ms, SD = 260ms) than in the within-task comparison (M = 585ms, SD = 211ms). However, this difference was only marginally significant (t(19) = 1.82, p = 0.084).

**Fig 3:**
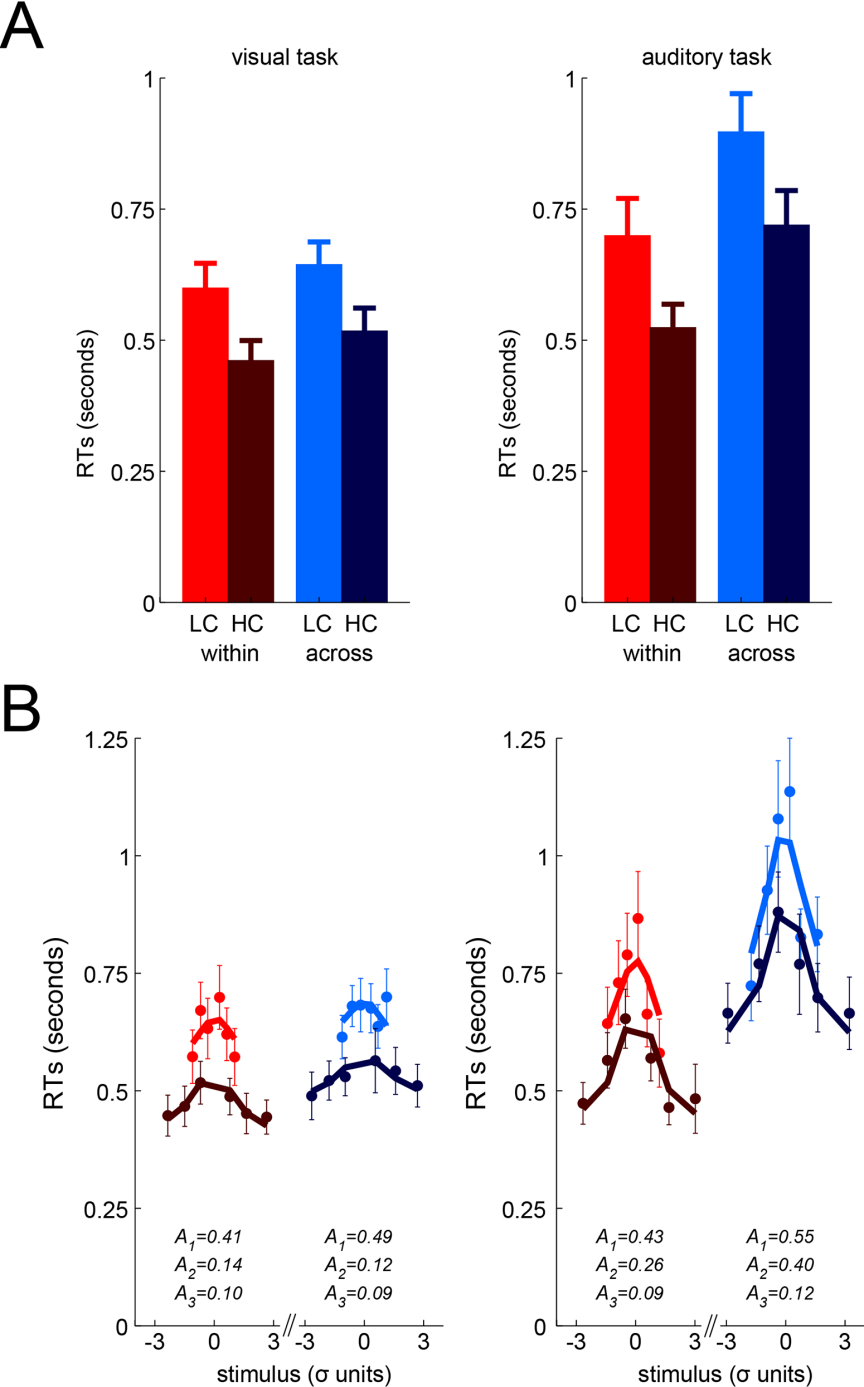
Response times analyses. (A) Median response times as a function of confidence (LC = low confidence in bright vs. HC = high confidence in dark), confidence comparison conditions (within-task in red vs. across-tasks comparison in blue), and task (visual in the left vs. auditory in the right panel). Bars represent the mean of observers with error bars for the SEM across observers. (B) Control analysis isolating the effect of stimulus on RTs. Median response times for bins of stimulus values (in units of standard-deviation on the psychometric curve, see text for details). Same color conventions as in (A). Dots and error bars represent mean and SEM across observers. Lines represent the average fitted RTs in each bin. The average values of the fitted parameters are indicated for each plot.

Median RTs for the perceptual tasks were submitted to an ANOVA with confidence (HC vs. LC) and comparison type (within vs. across) as within-participant factors (see Fig 3A), separately for the visual and auditory tasks. In both tasks, we found a main effect of confidence (visual: F(1,19) = 23.82, p<0.001), auditory: F(1,19) = 36.69, p<0.001), with faster responses when confidence was higher. In addition, there was a main effect of comparison type, with faster responses in the within-task condition relative to the across-task condition, although this effect was significant in the auditory task (F(1,19) = 20.03, p<0.001) and only marginal in the visual task (visual: F(1,19) = 3.34, p = 0.083), possibly because our paradigm involved visual instructions which may have cause an asymmetry between the visual and auditory tasks. There was no interaction between confidence and comparison type (visual: F(1,19) = 0.49, p = 0.49; auditory: F(1,19) = 0.002, p = 0.97).

Our analyses of RTs indicated that participants were faster when more confident, and slower in across-task blocks. In a more detailed analysis, we ensured that these effects were not confounded with the variation in stimulus difficulty (e.g. easier stimuli would lead to both faster responses and greater confidence). To do so, we modeled for each participant the relation between the stimulus values and response times in each condition to remove the influence of stimuli on RTs (see Methods and Fig 3B). This analysis provides 3 parameters in each task x comparison type, that correspond to 1) the intercept, 2) the amplitude of the stimulus influence on RTs and 3) the residual variation of RTs with confidence. The first and last parameters are of interest here, and were analyzed at the group level using ANOVAs and T-tests across participants. Importantly, the third parameter (confidence effect) was positive in all conditions (t-tests against zero: all p<0.05), indicating that high confidence responses were faster, even when taking the stimulus effect into account. This parameter was also unaffected by the task, the type of confidence comparison (within vs. across), or their interaction (all F<0.26, p>0.6). In other words, the speed-up of RTs with confidence was virtually identical in all conditions. In addition, the intercept parameter exhibited a main effect of type of confidence comparison which confirmed that participants were slower in across-task blocks relative to within-task blocks (visual: 75ms, t(19) = 2.27, p = 0.035; auditory: 116ms, t(19) = 2.14, p = 0.046), as found in the previous analysis.

## Discussion

The present study provides evidence that observers can compare their confidence across visual and auditory tasks without incurring any cost regarding metacognitive ability, relative to comparisons within the same task. In other words, the information provided by the visual and auditory systems, once evaluated in terms of decision confidence, can be converted without additional error to an abstract format that enables generic comparisons across the two tasks. These findings confirmed and extended our previous results showing that such comparisons can be made between two visual tasks [13]. Our study thus provides empirical evidence consistent with the idea that confidence is represented on a common scale between visual and auditory tasks, and substantiates the idea that confidence can serve as a common currency for judging our own decisions.

In addition to our analyses of metacognitive ability, we found that response times (RTs) were lengthened in across-task blocks compared to within-task blocks. For perceptual RTs, this time cost relates to the classic task-switching phenomenon (see e.g. [15]). This lengthening of RTs when the task changes occurs even when the switching of tasks is predictable [17], and both within and across modalities [18], which may explain its occurrence in the present experiment and in our previous work within the visual modality [13]. Because metacognitive RTs were marginally longer in across-task blocks, one could argue that there is a small time cost for converting confidence to a common format between the two tasks. We offer another interpretation by speculating that the continuous task-switching in across-task blocks induced some fatigue in participants. Interestingly, this fatigue would lengthen both perceptual and confidence RTs, and indeed we found that these two lengthenings were correlated across participants (r = 0.66, p = 0.0014), supporting this interpretation. In any case, despite the potential costs on response latencies, there seems to be no cost in terms of metacognitive sensitivity: participants were able to use a common scale for confidence between the visual and auditory tasks, with no additional errors.

The existence of a common scale for confidence when judging distinct perceptual dimensions might be helpful when deliberating about a multidimensional perceptual stimulus. For instance, to evaluate the probability that a mango is ripe, one might consider both its color (a red mango is likely to be ripe) and its size (a big one is likely to be ripe). Having a common currency for confidence facilitates the combination of information between these two dimensions, which might help optimizing choices. Future research might also investigate the use of a common scale for confidence when judging the same attribute (e.g. size) via different cues (e.g. haptic and visual information).

In collective decisions too, the use of confidence as a common currency when exchanging and combining information could help participants in achieving better decisions. Indeed, empirical evidence shows that confidence partly determines the influences of individual members during group deliberation [19], and that communicating choice and confidence information improves the accuracy of collective choices [20], at least in situations where most participants are correct [21].

Confidence appears thus to be a natural common currency for describing our own decisions, that is comparable across tasks and can be communicated across individuals. From this perspective, it is in principle compatible with the utility scale, and confidence and utility could be combined to form subjective expected utilities, as prescribed by theoretical models of optimal behaviour [2]. In terms of the brain mechanisms involved, it could be envisioned that because both confidence and utility have “common currency properties” and need to be integrated to compute subjective utilities, they might be represented within the same brain regions. In other words, one could expect the ventro-medial prefrontal cortex, which has been associated with the coding of subjective values in a large number of studies (e.g. [6], for a review see [7]), to be sensitive to confidence. Recent empirical work seems to confirm this hypothesis [22].

## Supporting Information

S1 File data.zip contains the data, presented as a matlab structure format and as a csv file. (ZIP)
